# Supplementary material for: Gender Expression, Weight Status, and Risk of Experiencing Eating Disorders among Gender-Diverse Adults Assigned Male at Birth in Bangkok, Thailand
Source: Nutrients. 2023 Aug 24;15(17):3700. doi: 10.3390/nu15173700 (PMC10489815; doi:10.3390/nu15173700)
Supplement: Supplementary file 1 [file nutrients-15-03700-s001.zip › nutrients-2535485-supplementary.pdf]

Supplementary Table S1. Descriptive characteristics of Thai gender-diverse adults assigned male at birth according to data completeness (n=217)

Descriptive Characteristics	Data Completeness		Total (n=238)	p-value†
	Complete (n=217)	Missing (n=21)		
Age, years, mean (SD)	29.9 (6.19)	31.3 (6.16)	30.1 (6.19)	0.34‡
Birthplace, n (%)				0.26§
Bangkok	128 (59.0%)	15 (71.4%)	143 (60.1%)	
Other provinces	89 (41.0%)	6 (28.6%)	95 (39.9%)	
Income per month, n (%)				0.06§
Quartile 1 (21,500 THB¶ or less)	52 (24.0%)	10 (47.6%)	62 (26.1%)	
Quartile 2 (21,501 – 33,000 THB¶)	53 (24.4%)	2 (9.5%)	55 (23.1%)	
Quartile 3 (33,001 – 60,000 THB¶)	61 (28.1%)	3 (14.3%)	64 (26.9%)	
Quartile 4 (60,001 THB¶ or more)	51 (23.5%)	6 (28.6%)	57 (23.9%)	
Current Living status, n (%)				0.39§
Alone	92 (42.4%)	6 (28.6%)	98 (41.2%)	
With Friends/Family	92 (42.4%)	10 (47.6%)	102 (42.9%)	
With partner/couple	33 (15.2%)	5 (23.8%)	38 (16.0%)	
Religion, n (%)				0.06§
Atheist	142 (65.4%)	9 (42.9%)	151 (63.4%)	
Buddhist	69 (31.8%)	10 (47.6%)	79 (33.2%)	
Other religion	6 (2.8%)	2 (9.5%)	8 (3.4%)	
Educational attainment, n (%)				0.32§
Lower than bachelor's degree	147 (67.7%)	12 (57.1%)	159 (66.8%)	
Bachelor's degree or higher	70 (32.3%)	9 (42.9%)	79 (33.2%)	
Relationship Status, n (%)				0.77§
Single	131 (60.4%)	12 (57.1%)	143 (60.1%)	
Partnered/Married	86 (39.6%)	9 (42.9%)	95 (39.9%)	
Current Smoking Status, n (%)				0.88§
Yes	12 (5.5%)	1 (4.8%)	13 (5.5%)	
No	205 (94.5%)	20 (95.2%)	225 (94.5%)	
Current Alcohol Consumption, n (%)				0.94§
Yes	74 (34.1%)	7 (33.3%)	81 (34.0%)	
No	143 (65.9%)	14 (66.7%)	157 (66.0%)	
History of Mental Health Issues, n (%)				0.35§
Yes	26 (12.0%)	4 (19.0%)	30 (12.6%)	
No/unsure	191 (88.0%)	17 (81.0%)	208 (87.4%)	

†All comparisons were made between masculine vs. feminine/androgynous group; ‡Independent T-test p-value; §Chi-square p-value; ¶THB, Thai Baht.

Supplementary Table S2. Multivariable logistics regression to explore gender expression and weight status in association with the risk of experiencing eating disorders and extreme weight control behaviors in Thai gender-diverse adults assigned male at birth (n=217)

**Variables**	**EAT-26[‡] ≥ 12**	**EAT-26[‡] ≥ 20**	**Extreme Weight Control Behaviors**
**Gender Expression**			
Masculine	Ref.	Ref.	Ref.
Feminine/ androgynous	0.49 (0.27, 0.88)	0.33 (0.12, 0.91)	0.70 (0.39, 1.26)
**Current BMI[†]**	1.01 (0.95, 1.07)	1.00 (0.92, 1.09)	1.07 (1.01, 1.14)
**BMI[†] discrepancy[§]**	1.04 (0.95, 1.13)	1.03 (0.92, 1.15)	1.13 (1.03, 1.24)

[†]BMI, body mass index; [‡]EAT-26, Eating Attitute Test-26; [§]BMI discrepancy is defined as the difference between current and ideal BMI ($BMI_{current}$ – BMI_{ideal}); Results are shown as odds ratio (95% confidence interval).
